# Update on Metabolic Bariatric Surgery for Morbidly Obese Adolescents

**DOI:** 10.3390/children8050372

**Published:** 2021-05-09

**Authors:** Holger Till, Oliver Mann, Georg Singer, Susann Weihrauch-Blüher

**Affiliations:** 1 Department of Pediatric and Adolescent Surgery, Medical University of Graz, 8036 Graz, Austria; georg.singer@medunigraz.at; 2 Department of General, Visceral and Thoracic Surgery, University Medical Center Hamburg-Eppendorf (UKE), 20246 Hamburg, Germany; omann@uke.de; 3 Clinic for Pediatrics I, Pediatric Endocrinology, University Hospital Halle (Saale), 06120 Halle (Saale), Germany; susann.weihrauch-blueher@uk-halle.de

**Keywords:** adolescence, childhood, obesity, metabolic and bariatric surgery, guidelines

## Abstract

Despite worldwide public attention and intense medical efforts, the prevalence of severe morbid obesity in children and adolescents is still rising. Similar to adults, excess adipose tissue triggers multiple immunological and metabolic pathways leading to serious co-morbidities such as impaired glucose tolerance or even type 2 diabetes (T2D), dyslipidemia, arterial hypertension, non-alcoholic fatty liver disease, and hyperuricemia. The management of severe childhood obesity requires a life-long multidisciplinary approach with a combination of lifestyle changes, nutrition, and medications. Standardized life-style intervention programs remain the first-line treatment for morbid obese children and adolescents, but unfortunately reveal limited long-term success. In such cases, metabolic bariatric surgery (MBS) has evolved from being a controversial issue to being included in distinct recommendations. According to the American Society for Metabolic and Bariatric Surgery (ASMBS) Pediatric Committee, indications for bariatric surgery in adolescence must follow very strict criteria. Adolescents with class II obesity (BMI > 120% of the 95th percentile) and a diagnosed co-morbidity or with class III obesity (BMI ≥ 140% of the 95th percentile) should be considered for MBS. These interventions represent high-risk operations, and adolescents should be treated in specialized, multidisciplinary high-volume obesity centers with long-term follow-up programs. The Roux-en-Y gastric bypass (RYGB) remains the gold standard of all malabsorptive procedures. Laparoscopic sleeve gastrectomy (LSG), which the authors pioneered as a stand-alone procedure in morbidly obese adolescents in 2008, has become the most commonly performed operation in morbidly obese adolescents at present. Recent literature proves that MBS is safe and effective in morbidly obese adolescents. Mid-term data have revealed significant improvement or even resolution of major co-morbidities. Thus, MBS for the treatment of morbidly obese adolescents has evolved from being a controversial issue to being included in distinct recommendations by several medical societies as a therapeutic strategy to reduce severe co-morbidities potentially causing end-organ damage in adulthood.

## 1. Introduction

Despite worldwide public attention and intense medical efforts, the pandemic of severely morbid obesity in children and adolescents has continued over the last decades [1]. Similar to adults, excess adipose tissue triggers multiple immunological and metabolic pathways resulting in serious comorbidities including disturbed glucose metabolism leading to prediabetes or type 2 diabetes, non-alcoholic fatty liver disease, renal disease, hyperlipidemia, and hyperuricemia [2,3,4]. In addition, adolescents with severe obesity seem to present a pronounced risk for developing major cardiovascular morbidities in early adulthood [5]. Furthermore, orthopedic problems as well as obstructive sleep apnea syndrome often already exist in these children [6]. Obese children and adolescents also suffer from psychological and social sequelae such as depression, stigmatization, bullying, social isolation, and poor self-esteem [7].

If left untreated, obesity in childhood and adolescence has serious long-term medical and socioeconomic consequences, as it often persists into adulthood, including an increased risk to develop malignant diseases [8,9].

The management of severe childhood obesity requires a life-long multidisciplinary approach with a combination of lifestyle changes, nutrition, medications, and metabolic bariatric surgery (MBS) in selected cases. In children, overweight is classified as having a BMI ≥ 90th and < 97th percentile, obesity as having a BMI ≥ 97th and < 99.5th percentile, and extreme (morbid) obesity as having s BMI ≥ 99.5th percentile. Conservative lifestyle intervention programs remain the first-line treatment, but unfortunately reveal limited long-term success [10]. On the other hand, similarly to adults, surgical procedures represent an effective option for morbidly obese adolescents to lose weight and treat their obesity-related co-morbidities [3,11,12]. To prepare well for MBS in adolescence [13], this review updates the current information about pre-operative evaluations, selection of procedures according to benefits and complications, as well as long-term effects on co-morbidities.

## 2. Indication for Metabolic Bariatric Surgery in Adolescents

The American Society for Metabolic and Bariatric Surgery (ASMBS) Pediatric Committee has recently updated their guidelines [3]. Adolescents with class II obesity and a diagnosed co-morbidity or with class III obesity should be considered for MBS [14].

Pratt et al. recently recommended treatment teams to conduct a thorough pre-operative evaluation including metabolic profiling but also to provide sufficient education about the surgical interventions and the postoperative lifestyle changes that will be necessary [14].

In Europe, indications for MBS in morbidly obese adolescents vary between countries. Nevertheless, the European Association for the Study of Obesity and the European Chapter of the International Federation for the Surgery of Obesity have agreed upon interdisciplinary European guidelines on MBS in adolescents in 2013 [15]. According to these guidelines, bariatric surgery can be considered in patients

1. with a BMI > 40 kg/m^2^ and at least one confirmed comorbidity;
2. following at least 6 months of organized weight-reducing attempts in a specialized center;
3. showing skeletal and developmental maturity;
4. capable to commit to comprehensive medical and psychological evaluation before and after surgery;
5. willing to participate in a post-operative multidisciplinary treatment program;
6. with the possibility to access surgery in a unit with specialist pediatric support (nursing, anesthesia, psychology, post-operative care).

Moreover, bariatric surgery can even be considered in patients with genetic syndromes such as Prader–Willi syndrome, but only after careful consideration by an expert medical, pediatric, and surgical team [15].

The German guideline published by the German Society for General and Visceral Surgery describe a BMI ≥ 35 kg/m^2^ and at least one diagnosed somatic and/or psychosocial comorbidity as indication for bariatric surgery in children and adolescents. In patients with a BMI ≥ 50 kg/m^2^, surgery can be considered even without any comorbidity. The patient, however, should have reached 95% of the predicted final height or Tanner stage IV. Before surgery, the adolescents should have undergone at least 6 months of unsuccessful conservative interdisciplinary lifestyle intervention programs [16].

Exclusion criteria for bariatric surgery in adolescents include:

- Severe psychiatric disorders (unstable psychosis, borderline personality, severe depression and personality disorders, active suicidality) and diagnosed eating disorders;
- Alcohol and/or drug abuse;
- Pregnancy (present or planned within 18 months after surgery);
- Inability of the patient to participate in a long-term interdisciplinary follow-up at the obesity center.

Immediately preoperatively, most multidisciplinary bariatric surgery programs nowadays recommend a protein-rich liquid diet to induce weight loss and decrease the mass of the liver size. Such conditioning improves surgical safety, operative times, and intraoperative blood loss [12].

Moreover, psychologists should examine the adolescents’ readiness and suitability for bariatric surgery. Amongst others, motivation, adherence, and the ability to understand pre- and postoperative requirements should be assessed [17].

When dealing with obese adolescents who may qualify for bariatric surgery, the role of the parents should not be underestimated because parental attitudes towards the weight status of their children may pose significant barriers to their consideration of treatment options [18]. It has been shown that most parents of overweight children fail to recognize that their child has a weight problem [19,20]. Moreover, in their telephone survey, Singh and Chernoguz found that only half of the parents with obese children concerned for their child’s future health would consider metabolic surgery, and most parents who would consider metabolic surgery would wait until their child was over age 18. However, provider counseling seems to significantly increase parents’ likelihood to consider bariatric surgery [18]. The authors concluded that “more detailed provider education on the current state of bariatric surgery in the treatment of severe adolescent obesity is necessary.” [18].

## 3. Surgical Principles and Methods

Despite a recent increase in the number of these surgeries, adolescents still constitute only a very small proportion of patients undergoing bariatric procedures. In the United States, adolescents constitute only 0.1% of all patients subjected to metabolic and bariatric procedures [21]. As a consequence, and according to the present guidelines, MBS should be performed in high-volume centers specialized in the medical and surgical treatment of morbid obesity at any age. Thus, it seems highly advisable for pediatric surgeons to join a high-volume center for adult bariatric surgery. In the US, the Metabolic and Bariatric Surgery Accreditation and Quality Improvement Program (MBSAQIP) requires for adolescent bariatric surgery that a children’s hospital conducting fewer than 25 stapling cases per year invites an MBSAQIP-verified bariatric surgeon on each case [14].

Pathophysiologically, a fundamental distinction is made between the so-called restrictive versus the malabsorptive procedures. Restrictive procedures “simply” tighten the stomach, for example, by placing an adjustable band around the fundus (laparoscopic adjustable gastric banding, LAGB). This maneuver creates a pouch and a small channel into the remaining stomach. The volume of food passing through the band may be adjusted by changing the diameter of the band. The continuity of the gastrointestinal tract remains intact.

The Roux-Y gastric bypass (RYGB) remains the “gold standard” of all malabsorptive procedures. Technically, the stomach is resected close to the gastroesophageal junction, leaving only a small pouch of approximately 20 mL. A loop of the small bowel is anastomosed to this gastric pouch (alimentary loop), while the rest of the stomach, duodenum, and the adjoining proximal small intestinal loops are initially excluded from the food route (biliodigestive loop). Further distally (100–170 cm), both loops are joined (common channel), allowing the absorption of food.

Sleeve gastrectomy (LSG, Laparoscopic Sleeve Gastrectomy) may serve both principles. Basically, the stomach is resected longitudinally, leaving a “restricted” volume of approx. 50–100 mL. This procedure was first recommended by the authors as a “stand-alone” technique for young adolescents in 2008 [22]. Metabolically, it is assumed that resection of the gastric fundus also extensively removes ghrelin production. Since ghrelin induces feelings of hunger in the central nervous system (arcuate nucleus), the removal of ghrelin-producing cells induces changes in the eating behavior [23,24,25]. Valid studies have shown that it is almost as effective as RYGB in inducing weight loss and improving co-morbidities [26]. Thus, LSG has gained general acceptance and has become the most commonly performed MBS procedure in adolescents [3].

## 4. Results of MBS in Adolescents

### 4.1. Effects on Weight Status

The American Teen-LABS Consortium published a prospective randomized study in 2016 comparing RYGB versus LSG versus LAGB. In summary, the data revealed a sustainable weight reduction of 27% in 242 adolescents (161 RYGB, 67 LSG, and 14 LAGB) after 3 years. There were no significant differences between RYBG and LSG (mean weight loss: RYGB 28% and LSG 26%). The adjustable gastric band (LAGB) performed worst (8%) [26].

Previous studies support these findings. A systematic review article and meta-analysis on weight loss after bariatric surgery in adolescence has shown that the mean BMI difference between the initial examination at the time of the operation and the examination after one year was −13.5 kg/m^2^ (95% confidence interval −14.1 to −11.9 kg/m^2^) [27]. Weight loss was the greatest after RYGB and the least after treatment with an adjustable gastric band (AGB) [27].

In a recently published report, 50 adolescents receiving Roux-en-Y gastric bypass were assessed 1 year and between 5 and 12 years postoperatively [28]. Compared to a non-surgical group, the BMI of these patients significantly declined from baseline during one year. Despite some regain, however, the weight loss was largely maintained 5–12 years postoperatively [28].

Overall, these results should be viewed cautiously, and patients should be followed up carefully, since long-term studies of adults have shown that many MBS patients gain weight again after 3 to 10 years, regardless of the surgical method used [29]. Therefore, long-term follow-up examinations after bariatric surgery in adolescents are imperative and should be performed in specialized centers with a pediatric surgeon, a pediatrician/pediatric endocrinologist, a psychologist, and an ecotrophologist. Moreover, since the treatment of obesity in childhood has been shown to be more effective with a family-based approach, when both parents and children are targets of behavioral modification [30], the role of family and social environment regarding long-term outcome following bariatric surgery should be more closely assessed in future studies.

### 4.2. Effects on Pre-Existing Comorbidities

In adulthood, LSG and LAGB have revealed similar efficacy at one-year and three-year follow-up with regard to weight loss and improvement of comorbidities [24].

Initial results from adolescents also showed a significant improvement of pre-existing type 2 diabetes, obstructive sleep apnea syndrome, bronchial asthma, non-alcoholic fatty liver disease, and dyslipidemia [31,32]. The Teen LABS study described an improvement or complete disappearance of pre-existing type 2 diabetes in 90%, dyslipidemia in 66%, arterial hypertension in 74%, and impaired kidney function in 86% of adolescent patients [26]. In the follow-up of Adolescent Bariatric Surgery 5+ (FABS-5+) study, it has been shown that the values of all lipids, except for total cholesterol, eight years after gastric bypass significantly improved compared to their preoperative values [33]. Ryder et al. demonstrated that in adolescents with severe obesity undergoing bariatric/metabolic surgery, the risk of hard cardiovascular endpoints was extensively improved later in life [5]. Furthermore, these data suggest that surgery in adolescents with severe obesity is cost-effective by preventing later cardiovascular events including early cardiovascular deaths [5]. These findings are comparable to long-term outcome data in adults after MBS [34,35,36,37].

In addition, improvements in insulin sensitivity and glucose homeostasis, a decrease of serum free fatty acid levels, an increase of the adiponectin/leptin ratio, and a decrease of interleukin-6 and C-reactive protein have been reported [38,39,40,41].

Regarding psychological outcomes, an observational study has revealed that at 2 years, approximately one in three adolescents was symptomatic for a psychopathology and that the majority of adolescents maintained their symptomatic or non-symptomatic status from baseline to 24 months post-baseline [42]. Moreover, in an examination of mental health of 82 adolescents at baseline, one and two years after following laparoscopic gastric bypass, 20% of the patients had poor mental health [43]. An increased number of anxiety- and depression-related symptoms and worse mental health at baseline significantly predicted poor mental health two years postoperatively. In a recent study, Zeller and coworkers have shown that bariatric surgery in adolescence does not change the risk of suicidal thoughts and behavior during the initial 4 years after surgery [44]. Suicide risks present before surgery persisted and also newly emerged in a subgroup with poorer psychosocial health [44]. These findings were confirmed by a study by McPhee et al. who found that in a noteworthy subset of adolescents receiving bariatric surgery, suicidal ideation can be found preoperatively and postoperatively [45]. Another interesting study performed in the USA found that the pregnancy rate for adolescent females undergoing bariatric surgery was greater than that of age- and race-matched females [46]. Potential factors that may have contributed to this increased rates remain unclear, but may include poor adherence to contraceptive therapy and psychosocial factors [46]. Taken together, it is of great importance that children and adolescents are assessed psychologically and screened for suicidal thoughts and behavior before as well as after bariatric surgery.

### 4.3. Complications

MBS consists of high-risk operations and may induce distinct surgical complications. Basically, there are more “technical” complications with LAGB. In up to 30% of all cases, dislocations of the band or erosions into the stomach may be encountered, requiring revisional surgery. Paulus et al. have determined the complication rate in bariatric surgery in adolescence in their last systematic review: complications occurred in around 11% of patients who received an AGB, including dislodgement of the band and port revisions [47]. Due to the high rate of long-term revisional surgery, in adults, AGB has become a very rare procedure in the last years.

In patients subjected to malabsorptive techniques, the “principle may become a problem”, meaning that malnutrition, vitamin deficiencies, or electrolyte imbalances may occur in the long term. The most important and most frequent long-term complication in adolescents after bariatric surgery, however, are deficiency symptoms of vitamins or trace elements that occur after all three surgical procedures. Deficiency symptoms should therefore be recognized and corrected before the operation. The most common vitamin deficiency symptoms following RYGB are vitamin B12 deficiency, thiamine deficiency, and vitamin D deficiency [48].

The Teen-LABS study revealed that 37% of the patients had a vitamin D deficiency preoperatively and that this deficiency persisted in 43% of the patients three years after RYGB and SG. A pre-existing vitamin B12 deficiency worsened significantly 3 years postoperatively. Furthermore, 5% of the patients had an iron deficiency preoperatively, and three years postoperatively this rate was increased to 57% [26]. A life-long vitamin substitution after bariatric surgery is therefore recommended.

## 5. Discussion

The term metabolic bariatric surgery (MBS) claims that such procedures lead to weight loss by influencing metabolic pathways of energy homeostasis. Current studies in adults have shown that MBS can improve insulin resistance and secretion independently of weight loss by influencing gastrointestinal hormones. In some patients, complete remission of type 2 diabetes occurred just a few days after bariatric surgery, that is, before significant weight loss was recorded [49,50].

Such observations suggest that MBS directly influence the hormonal control of glucose balance [51]. Thus, these procedures represent an effective treatment approach for obesity-associated type 2 diabetes. The remission rate depends not only on the duration of the manifest diabetes or the time of the operation, but also on the surgical procedure and, most importantly, on the postoperative compliance of the patient with following nutritional recommendations [51]. Additionally, genetic factors play a yet not completely unraveled but significant role [52,53]. The highest remission rates in type 2 diabetes have been achieved after a Roux-en-Y gastric bypass (RYGB) and after biliopancreatic diversion with duodenal switch (BPD/DS). Remission rates ranging between 40% and 100% have been described for the different surgical procedures, with gastric bypass appearing to have higher remission rates than the purely restrictive procedures [49,51,54]. The remission rates in patients with type 2 diabetes reached an average of 76.8%:38% after gastric banding, 84% after RYGB, and 98% after BPD with or without duodenal switch [55]. Procedures like BPD with or without duodenal switch are reserved to a minority of (very sick) patients, mainly due to their potential long-term complications; and in children and adolescents, only extremely limited data exist [50,56,57].

MBS for the treatment of extreme obesity in children and adolescents has evolved from a controversial issue to being included in distinct recommendations [3]. Malabsorptive procedures remain the gold standard. Overall, the laparoscopic Roux-en-Y gastric bypass (LRYGB) has been the most commonly used method to date in extremely obese adolescents [58,59,60,61,62,63,64,65,66]. Historically, the laparoscopically adjustable gastric band (LAGB) has also been used frequently [59]. LSG has gained general acceptance and has become the most commonly operation performed in morbidly obese adolescents [3]. An analysis of the Metabolic and Bariatric Surgery Accreditation and Quality Improvement Program (MBSAQIP) database including patients following sleeve gastrectomy and gastric bypass procedures from 2015 to 2018 has, however, revealed that out of 760,076 patients, only 0.1% were adolescents, and the majority of those were female and white. Therefore, there may be a disparity in access to bariatric surgery among adolescents, particularly for racial and ethnic minorities [21].

Initial results after bariatric surgery in morbidly obese adolescents with metabolic comorbidities suggested that these procedures improved an impaired glucose metabolism and other cardiometabolic comorbidities even in this age group and could even be more effective than in adults [67,68,69]. Moreover, there are only few differences in the outcome of bariatric surgery (RYGB and SG) between younger and older adolescents, suggesting that younger adolescents with severe obesity should not be denied consideration for surgical therapy [70].

However, the majority of reports available to date on bariatric surgery in (children and) adolescents are, with the exception of the Teens LAB study, retrospective studies. Previous studies in extremely obese pediatric patients showed a significant benefit in the short-term follow-up. Long-term data are still pending. There are still no definitive therapy recommendations, in particular for the bariatric methods suitable for children and adolescents. The implementation of randomized controlled clinical trials is therefore essential, and metabolic surgery as well as follow-up examinations should thus only be performed in specialized centers, which offer a long-term interdisciplinary treatment program.

## 6. Summary and Outlook

MBS for the treatment of extreme obesity in children and adolescents has evolved from a controversial issue to being included in distinct recommendations. Treatment teams should conduct a thorough pre-operative evaluation including metabolic profiling but also provide sufficient education about the surgical interventions and the postoperative lifestyle changes that will be necessary. Indications for bariatric surgery in obese adolescents follow strict current guidelines. MBS should be carried out in high-volume centers specialized in the medical and surgical treatment of morbid obesity at any age. Long-term observation focusing on the course of comorbidities as well as potential metabolic complications seem absolutely mandatory for this age group. The American Society for Metabolic and Bariatric Surgery (ASMBS) Pediatric Committee recommends MBS should not be withheld from adolescents with severe co-morbidities to reduce the risk of end-organ damage.
